# Pride and adversity among nurses and physicians during the pandemic in two US healthcare systems: a mixed methods analysis

**DOI:** 10.1186/s12912-022-01075-x

**Published:** 2022-11-09

**Authors:** Igor Burstyn, Karyn Holt

**Affiliations:** 1 Department of Environmental and Occupational Health, Dornsife School of Public Health, Drexel University, 3215 Market Street, Nesbitt Hall, Room 640, 19104 Philadelphia, PA USA; 2 School of Nursing, the University of Nevada Las Vegas, Las Vegas, NV USA

## Abstract

**Background:**

Our aims were to examine themes of the most difficult or distressing events reported by healthcare workers during the first wave of COVID-19 pandemic in two US health care systems in order to identify common themes and then to relate them to both behavioral theory and measures of anxiety and depression.

**Methods:**

We conducted a cross-sectional survey of nurses and physicians during the early phases of the COVID-19 pandemic in the US. An emailed recruitment letter was sent, with about half choosing to supply open-ended responses relevant to thematic analysis. We measured symptoms of anxiety and depression separately, captured demographics, and asked two open-ended questions regarding events that were the most difficult or stressful, and reinforced pride. We reported descriptive statistics and coded thematic categories for their continuum “pride” and “distress” the factors related to fostering well-being according to the Self-Determination Theory.

**Results:**

Themes that emerged from these narratives were congruent with prediction of Self-Determination theory that autonomy-supportive experiences will foster pride, while autonomy-thwarting experiences will cause distress. Those who reported distressful events were more anxious and depressed compared to those who did not. Among those who reported incidences that reinforced pride in the profession, depression was rarer compared to those who did not. These trends were evident after allowing for medical history and other covariates in logistic regressions.

**Conclusion:**

Causal claims from our analysis should be made with caution due to the cross-sectional research design. Understanding perceptions of the pandemic by nurses and physicians may help identify and manage sources of distress, and suggest means of mitigating the risk of mental health distress through autonomy-supportive policies.

**Supplementary Information:**

The online version contains supplementary material available at 10.1186/s12912-022-01075-x.

Causal claims from our analysis should be made with caution due to the research design, a cross-sectional study design. Understanding of perceptions of the pandemic by nurses and physicians may help identify sources of distress and means of reinforcing pride in the professions, thereby helping nurses and physicians cope with disasters, and shape workplace policies during disasters that foster well-being.

## Background

Evidence is robust that nurses and physicians around the world suffered from mental health distress, including anxiety and depression, during the COVID-19 pandemic [1–6], especially in intensive care units [7]. However, the specific perceptions of the events by nurses and physicians related to these mental health challenges are poorly captured, which may hinder effective interventions. Our prior analysis of risk factors for anxiety and depression among nurses and physicians during the first wave of COVID-19 in two health care systems in the US, who are also subjects in this report, posited that concern about contracting COVID-19 was a correlate of both anxiety and depression, especially among those who experienced recent bouts of poor health [8]. Furthermore, the risk of anxiety and depression was reduced among those who felt competent using personal protective equipment and had access to it, reported few changes to working hours, and were surrounded by sufficient numbers of colleagues who were not seem as stressed. The expected support of immediate family and religious communities were protective. We did not delve into the specifics of experiences in the first report but only captured them on visual-analogue scales, thus potentially missing important features that contributed to mood disorders. We speculated that the “impact of work organization on anxiety and depression … may be related to the role of the safety climate or culture in moderating impact of the pandemic on work-induced mental health issues” [9–11], and that Self-Determination Theory (SDT) may offer a suitable framework for addressing these issues [8].

In this manuscript, thematic analysis of self-reported experiences of nurses and physicians during the first wave of COVID-19 is interpreted within the SDT framework [12]. According to the SDT, if changes are perceived as fostering a person’s sense of *autonomy* (e.g., having at least some latitude or input in the nature of changes), *competence* (e.g., leading to high level of professional performance), and *relatedness* (e.g., strengthening bond with colleagues and community at large), the people intrinsically cooperate with the change and perceive them as positive (experience wellness). On the other hand, if the psychological needs for autonomy, competence and relatedness are thwarted due to perceived coercive means employed to implement changes, then people become de-motivated and recognize changes as negative (experience ill-being). In the context of COVID-19 pandemic, there is evidence from longitudinal study that experience of autonomy-thwarting environment (i.e. frustration of basic psychological needs) among university students was the main predictor of depressive symptoms, after accounting for history of depression [13]. The frustration of psychological needs postulated by STD was also were related to risk of mood disorders in a longitudinal analysis of adults experiencing COVID-19 lockdown in Belgium [14]. The COVID-19 pandemic precipitated changes in healthcare that necessitated changes in how nurses and physicians lived and practiced their professions. The extent to which such changes were positively received and the consequent ease of their acceptance and adoption, can be related to the perception of these changes as either autonomy-supportive or coercive. Although we did not formally access whether nurses and physicians experienced an autonomy-supportive vs. coercive workplace climate during implementation of changes resulting from COVID-19 pandemic, we can document whether their most difficult experiences (ill-being) correspond to the threats to their senses of autonomy, competence, and relatedness. Conversely, we can document whether their experiences that instilled or affirmed pride in their profession (wellness) corresponded to experiences that reinforced their autonomy, competence, and relatedness. Thus, although we are limited by not having employed psychometric scales typically used in evaluation of SDT (e.g., Work Climate and Problem at Work questionnaires), we have mapped themes identified in the narratives to psychological needs postulated by SDT.

We aimed to identify common themes among the most difficult or distressing events as well as experiences that instilled or reinforced a sense of pride reported by nurses and physicians and relate them to anxiety and depression during the first wave of COVID-19 pandemic in two US health care systems, one in the Northeast, the other in the West.

## Methods and materials

Our project received ethics approval from the Institutional Review Boards of the respective institutions.

### Study design and data collection

Details of the survey methodology and participating healthcare systems can be found in our earlier publication; participants were assured that their responses will remain anonymous [8]. We conducted a cross-sectional survey of all physicians and nurses employed and contracted by a medical center in Pennsylvania (TH) and in a Medical Center in the West(UMC), and licensed to practice in these states, corresponding to the early phases of the COVID-19 pandemic in the US. We recruited through Health Systems’ employee databases, distributing the invitation to enroll in the study and subsequent reminders by email with links to online surveys. Participants meeting the inclusion criteria received the recruiting email, clicked on a link to the study description and consent information in the recruitment email, and then to link to the actual Qualtrics survey. Participation in the survey indicated consent and participants responded to the open-ended questions and multiple choice questions via an online Qualtrics survey. Participation was voluntary, without reward for participation, and confidential. On June 3, 2020, we distributed invitations to TH survey to 203 advanced nurse practitioners and 4,336 registered nurses; at the same time, we distributed invitation to TH survey aimed at physicians to 2,496 active medical staff and 204 physician assistants. On September 9, 2020, we distributed invitations to the UMC version of the survey to 1,518 registered nurses and nurse practitioners, and 1,186 physicians.

We measured symptoms of anxiety and depression separately via a well-established the Hospital Anxiety and Depression Scale (HADS); scores of equal to or above 11 (range 0–21) indicate presence of these conditions (“case”) but are not clinical diagnoses [15, 16]. We recorded age, marital status, gender, children under 18 years of age living at home, profession (nurse or physician), healthcare system, history of anxiety and depression prior to the pandemic (and evidence of exacerbation requiring treatment a year before the pandemic), report of positive COVID-19 test, whether respondent “have you had an episode when you have been unwell for two or more consecutive days” (whether or not they reported for duty) since the start of the pandemic, and two open-ended questions regarding (a) “What has been the most difficult or stressful event you have had to deal with?” and (b) “What has been the event that has most reinforced your pride in your professional behaviour?”.

### Analysis

The open-ended questions were independently coded into themes developed by the authors; disagreement on which theme a response belonged to was resolved by counting partial agreement as agreement, given that overall, there was a high degree of concordance in coding (Gwet’s AC1 at least 0.8 for each theme); each open-ended response could belong to more than one theme. The authors classified themes in terms of their apparent relationship to factors in SDT that are hypothesized to either promote (themes of pride) or thwart (difficult or stressful events) intrinsic motivation.

We conducted exploratory principal components analysis on the measured mood disorders via HADS, history of anxiety and depression (and their treatment a year prior to the pandemic), having reported a distressing event or an event that reinforced pride in the profession. We determined the number of interpreted principal components using scree plots. We examined the association of “cases” of anxiety and depression in relation to themes from coded open-ended responses via multivariable logistic regression models, adjusting for demographics and health factors described above, as well as controlling for the profession and healthcare system. These yielded odds ratios (OR) and 95% confidence intervals (CI). Only effect estimates with p ≤ 0.2 were interpreted. Heterogeneity of effects was evaluated using Wald-style test [17]. All statistical calculations were performed in SAS v 9.4 (SAS Institute, Cary, NC).

## Results

### Descriptive statistics

We recruited 1,124 nurses and physicians for the study, most from TH (803 nurses, 174 physicians) rather than UMC (71 nurses, 74 physicians). More than a half, 621, shared the most distressing experiences with the rest not responding or indicating that none were applicable; physicians at UMC were the outliers with the lowest rate of report at 17 (22%). Almost a half, 510, reported some experiences related to reinforcing pride in their professions, with the physicians from UMC being an outlier (11 reports, 14%). Just over a third, 443, reported both stressful events and those that instilled pride in their professions.

Nurses were predominantly female (81%), White (89%), married or divorced (65%), aged 44 years on average (range 21–70), almost half had children under 18 years of age living at home (45%). Among nurses, 225 reported to have felt unwell since start of the pandemic and 16 reported a positive test for COVID-19; 45% had history of anxiety or depression with 20% reporting treatment a year before the onset of the pandemic.

There were more males among physicians (56%); they were more likely to be White (73%), married or divorced (68%), aged 50 years on average (range 24–75), almost half had children under 18 years of age living at home (49%). Among physicians, 43 reported to have felt unwell since start of the pandemic and 4 reported a positive test for COVID-19; 26% had history of anxiety or depression with 10% reporting treatment a year before the onset of the pandemic.

Exploratory principal components analysis suggests that there are four independent groups of nurses and physicians, accounting for 80% of common variance (details in Supplemental Material A). There is a group with history of anxiety and depression that was also symptomatic during the pandemic who tended not to report either distressing or proudful experiences. The second group reported both distressing and proud events but had neither history nor current symptoms of anxiety and depression. The third group was anxious and depressed at time of survey but had no history of these conditions and did not report any distressing or proudful experiences. The fourth group was characterized by both distressing experiences and being a case of depression, but low on anxiety scale and with no history of mood disorders. There results suggest that (a) persons with history of mood disorders are not the ones who are most likely to report events that either distressed or reinforced pride (groups 1 and 2) and (b) the history of mood disorders is not the sole driver of experiencing symptoms of anxiety and depression during the pandemic (groups 3 and 4).

### Risk of anxiety and depression

Among nurses and physicians who reported distressful events, the rate of cases of anxiety was higher (33%, 202/607) than among those who did not (25%, 65/262) and the rate of cases of depression was higher (12%, 70/607) than among those who did not (9%, 23/262). Among those who reported events reinforcing pride in the profession, the rate of anxiety was elevated (32%, 161/501) compared to those who did not (29%, 106/368) while the rate of depression was reduced (10%, 50/501) compared to those who did not (12%, 43/368). There was no evidence of multiplicative interaction between the reports of proudful and distressing experiences on the odds of either anxiety or depression. We observed the evidence of distressing experiences elevating the odds of anxiety: OR 1.39 (95% CI: 0.96, 2.00) after accounting for healthcare system and profession.

For depression, we observed evidence of *increased odds* with report of distressing experiences (OR 1.48; 95%CI: 0.85, 2.55) and *decreased odds* with reports of events that instilled pride in the profession (OR 0.71; 95%CI: 0.44, 1.14), after accounting for healthcare system and profession. The null hypothesis test for heterogeneity for the above two effect estimates yielded p = 0.02.

The effect estimates for the above-mentioned logistic regressions models were not materially different after adjustment for other covariates.

Associations of specific themes of distressing experiences with anxiety and depression are illustrated in Table 1. The most reported distressing experiences related to fear of infection (15%), concerns about use of and access to personal protective equipment (14%), lack of transparency in communications and administrative support (14%), and uncertainty (11%). When all factors were considered together, including adjusting for experiences that reinforced pride, the odds of anxiety was elevated among those who expressed worries about infecting immediate family (OR: 1.75; 95%CI: 0.89, 3.44), were dissatisfied with standards of patient care (OR: 3.09; 95%CI: 1.32, 7.24), and struggled with transparency of communications (OR: 1.50; 95%CI: 0.98, 2.29). Challenges with transparency of communication were also linked to elevated odds of depression (OR: 1.52; 95%CI: 0.85, 2.74). Furthermore, aggression from patients or their families (OR: 2.94; 95%CI: 0.66, 13.01) and feeling angry (OR: 2.66; 95%CI: 0.64, 11.07) where independently associated with depression after accounting for other factors.

**Table 1:** Distribution of responses of experiences related to most difficult experiences among 1124 physicians and nurses in relation the psychological needs from self-determination theory (STD) they undermined, and HADS scores > 10 (cases of anxiety and depression) among 869 with complete data

Psychological need undermined	Theme coded	All records		Persons with complete data				
					**Anxiety case**		**Depression case**	
		**N**	**%**		**N**	**%**	**N**	**%**
Competence & Autonomy	Change in work and work effort	61	5.4	56	18	32	8	14
Competence & Autonomy	Concerns about PPE access and use	161	14.3	160	51	32	19	12
Relatedness	Social stigma due to work in healthcare	5	0.4	5	2	40	1	20
Competences & Relatedness	Worry about infecting family	59	5.2	58	26	45	8	14
Competence & Relatedness	Aggression of patients or their families	13	1.2	13	4	31	3	23
Competences & Relatedness	Self-expressed anger	18	1.6	17	7	41	5	29
Autonomy	Change in schooling: self or child	7	0.6	7	4	57	1	14
Autonomy	Childcare shortage	30	2.7	30	14	47	3	10
Competence & Autonomy	Testing for COVID-19 concerns	29	2.6	29	5	17	2	7
Relatedness	Death in the family or a friend	10	0.9	10	2	20	1	10
Competence	Death of patient	89	7.9	86	37	43	12	14
Competence	Guilt over perceived substandard patient care	29	2.6	29	15	52	4	14
Autonomy & Relatedness	Lack of transparency in workplace communications and administrative support	157	14.0	153	57	37	23	15
Autonomy	Loss of income: self or family	87	7.7	86	23	27	8	9
Relatedness	Media coverage of the pandemic	10	0.9	9	2	22	0	0
Relatedness	Overstated risk of COVID-19 by others	5	0.4	5	0	0	0	0
Relatedness	Denial of severity of COVID-19 by others	8	0.7	8	2	25	2	25
Competence & Autonomy	Fear of infection and unavoidable proximity to strangers	171	15.2	165	53	32	20	12
Relatedness	Social isolation due to pandemic mitigation measures	62	5.5	62	21	34	6	10
Relatedness	Witnessing distressed colleagues	37	3.3	37	12	32	5	14
Relatedness & Competence	Training staff under changing policies	47	4.2	44	13	30	5	13
Autonomy & Competence	Uncertainty	128	11.4	125	46	37	12	10

Associations of specific themes of experiences that instilled or reinforced pride with anxiety and depression are illustrated in Table 2. The most reported proud experiences was high quality of care provided (19%), teamwork (17%), and composure under stress (13%). When all factors were considered together, including adjusting for difficult experiences, the odds of anxiety was reduced among those who expressed pride about technological sophistication of care (OR: 0.38; 95%CI: 0.10, 1.41), altruism (OR: 0.65; 95%CI: 0.35, 1.21), and not becoming infected (OR: 0.30; 95%CI: 0.09, 0.97). Support from community was independently associated with reduced odds of depression after accounting for other factors (OR: 0.47; 95%CI: 0.16, 1.34). Those who were proud of volunteering for hazardous tasks were at an elevated odds of both anxiety (OR: 1.70; 95%CI: 0.81, 3.55) and depression (OR: 2.47; 95%CI: 0.97, 6.29), after accounting for other covariates considered in our analysis.

**Table 2:** Distribution of responses of experiences related to pride in the profession among 1124 physicians and nurses in relation the psychological needs from self-determination theory they reflect and foster

Psychological needs fostered	Theme coded	All records		Persons with complete data				
					**Anxiety** **case**		**Depression** **case**	
		**N**	**%**		**N**	**%**	**N**	**%**
Autonomy	Adaptability and flexibility (self or colleagues)	102	9.1	102	35	34	8	8
Autonomy and Competence	Technological sophistication (inventiveness) in care for patients	17	1.5	17	4	24	1	6
Autonomy and Relatedness	Volunteered for hazardous tasks	63	5.6	61	22	36	10	16
Autonomy and Relatedness	Altruism	74	6.6	74	21	28	10	14
Competence	Composure under stress	151	13.4	150	52	35	15	10
	Comforting dying patient	19	1.7	19	10	53	3	16
	Not infected after exposure	22	2.0	22	5	23	1	5
	Patients survive, quality of care	216	19.2	212	66	31	21	10
Relatedness	Community outreach by oneself	19	1.7	19	8	42	3	16
	Support from community	64	5.7	63	20	32	5	8
	Support from leadership	43	3.8	43	12	28	2	5
	Teamwork	196	17.4	189	61	32	18	10
	Thanks from patients	42	3.7	42	17	40	6	14

### Themes of “What has been the most difficult or stressful event you have had to deal with?”

Frequencies and names of the coded themes among difficult or stressful events since the start of the pandemic are captured in Table 1. They also indicate our perceived association of these themes with autonomy-thwarting factors. The meaning of the coded themes is illustrated below using the respondents’ own words (more fully presented in Supplemental Material B).

#### Change in work or work-effort

Changes in working conditions precipitated by the pandemic were stressful for most. The sheer increase in volume and pace of work was commonly mentioned: “… days were non stop.” On the other side of the spectrum, some changed involved “reducing the work force due to plummeting volumes”. For others, changes involved alterating the nature of work: “Being pulled to the ICU to care for Covid-19 patients without a full orientation and changing my schedule from day shift to evening and night. “ For some, changes involved performing unfamiliar duties, while potentially endangering the usual (non-COVID-19) patients.

When such changes in the nature of work combined with poor communications and childcare obligations, difficult situations ensued: “Communication about daily changes and schedule was also lacking. I also have three children acclimating to on line school and was not always available to assist them with my schedule changes.”

Disruption of patient care not related to COVID-19 appeared challenging for those who were unaccustomed to not having answers for their patients. When change in work involved perceived reduction in quality of patient care, this proved ”a challenge”: “Not being able to offer touch, see facial expressions, or give a hugs.”

Even if the type and patterns of work did not appear to materially change, the extra effort performing these duties during the pandemic-induced procedures proved a source of most notable stress for some, especially when dealing with critically ill patients and deaths: “We had multiple deaths each week and docs were more intense and demanding than usual causing me more stress to the point of crying.” Work effort increased, causing distress, in cases where patients needed extra mental health support.

#### Economic insecurity, cancelled leaves

For some respondents, threats of loss of income to themselves or to colleagues proved demoralizing, a contradiction between messaging about the essential nature of work by healthcare workers during the pandemic and the reality of economic conditions that restricted their practice. Some loss of income was seen as consequence of poor planning, causing needless increase in work effort. The issues related to change in policies affecting personal time off were upsetting to many, seen as means to economize.

#### Unavoidable exposure to infected persons and fear of infection

Some nurses and physicians were distressed by their own “irrational fear of other” and “wondering if [they] will contract the virus no matter how careful”. Experiencing and anticipating risk of infection was reported among some of the most difficult experiences, with the underlying sense that infection control was directed by others, placing one’s family at risk: “I never-ever declined an assignment and lived in fear of infecting my family.” Concerns about testing for COVID-19 revolved around several themes, including “receiving patients … that were not tested and later turn out positive”, “false negative tests”, “patients lying during screening to reach face to face interaction with a provider”, “worrying about infecting others because I was not tested”, and “having … symptoms and waiting for my testing”.

Given that “social distancing” was one of the proclaimed means of infection control, when this was not enabled due to hospital policies, this resulted in frustration, likely fueled by a sense of powerlessness to avoid situations perceived as placing one at risk: “All doors are locked and we’re supposed to be social distancing, but here we stand like a herd of cattle, at the few entrances to wait to get our temps taken.”

Becoming ill or exposed during the pandemic was recounted as one of the most difficult experiences, with the concerns centering on how this affected one’s family: “I contracted the disease and was very ill for 2 weeks, requiring a hospitalization. most stressed about how this affected my family”. When family members did contract COVID-19, a difficult situation ensured, especially when there appeared to be no additional support during the crisis: “My [spouse] was gravely ill with COVID and I had to care for both him and my children AND then return to work to care for my patients.“

#### Personal protective equipment (PPE) and other infection control challenges

A matter closely related to risk of infection due to unavoidable contact with infected patients was the availability and utilization of PPE. Even possibly appropriate measures were a source of distress, like “constant” “… mask fit testing” and “… disinfecting”,, and “… putting the same dirty mask back onto my face every time disgusts me.” Concerns about inadequate PPE were naturally intermingled with concern about infection, causing reports of distress.

Some reported difficulties that arose from trying to elicit collaboration of patients with the infection control measures. For others, the concerns arose from the PPE and infection control measures adversely affecting communication that is integral to quality care: “The room has a huge loud fan for negative pressure you have a mask and a face shield on so not only can they [patients] not hear you at all but they can’t even read your lips.”

The effort involved in using PPE proved very onerous for many, including “working 13 hours in N95 causing severe SOB, and dizziness”. A common related source of distress arose from having to re-use PPE, both due to discomfort, fear of shortages of PPE, and knowledge that this is leads to sub-optimal protection (i.e., against PPE training).

For some, the access to PPE was the major concern, combined with perceived lack of preparedness of the healthcare system, and implied disregard for patient and staff safety: “The lack of adequate PPE putting my friends and coworkers at risk due to poor planning.”

A particularly difficult situations arose when it appeared that management refused to share the burden of risk with frontline workers who lacked PPE: “The [leadership team] let us go in and get contaminated but stayed in the hallway and watched.“.

#### Concerns about health of oneself and others, not related to contracting COVID-19

Being unable to receive proper medical care due to infection control protocols in place proved difficult for some: “I had a scheduled appointment with my [specialist] and it was very upsetting and stressful that they wanted to reschedule it due to the sole fact that I am … working with COVID patients.” Some noted “lack of assistance getting access to mental health”. For a few, “unexpected death” of a family member during the pandemic overshadowed any other difficulties.

#### Death of patients

For some nurses and physicians, the sheer rate of mortality was overwhelming: “5 deaths in one shift.” The loneliness of dying patients due to infection control protocols that isolated them from their families was a common “heartbreaking” theme: “Seeing nurses so defeated in having to facetime with families to help the loved ones say goodbye….”.

Some reported struggling with the sense of failure and futility of effort in trying to avert death of the COVID-19 patients, combined with belief that such patients could have received better care, including emotional support, even if they could not be saved: “Very difficult to watch a patient suffocate slowly over weeks only to die despite our best efforts. No family is allowed to visit. Horribly sad.” Even some veterans of the field who “can handle almost anything” found they “cried everyday” when dealing with dying COVID-19 patients.

Some choices that nurses and physicians had to make, precipitated by infection control protocols, appear to be the stuff of ethical nightmares: “Patient likely going to die and having to limit which son will be able to remain.” A recurring, experience appeared to have been related to lapse in ethics of care due to lack of communication with families of patients: “… the DNR/AND is not honored.”

#### Perceived lapse (guilt over) in standards of patient care

Some nurses and physicians were burdened with “a lot of guilt” over belief that they did not provide the usual level of care. Those who had work hours reduced reported that “not being able to provide nursing care in a pandemic has caused feelings of worthlessness.” For some, concerns about quality of patient care appear to have been aggravated by breakdown in teamwork and fairness in allocation of responsibilities across a team.

#### Social isolation

For some, the most difficult experiences of the pandemic arose from the loss of the usual social contacts. In many cases this was related to self-isolation for fear of spread of infection to family members at high risk from the virus, especially when combined with recognized mental health difficulties: “Dealing with my personal depression and anxiety, self isolation, not being able to see my mother in her SNF [skilled nursing facility]”. Isolation from family and wider community made coping with the pandemic more challenging for some, precisely because family, community and friends were the usual sources of support. For those who rely on co-workers for support and companionship, having to stay away from work for fear of spreading infection to vulnerable family members was distressing: “Staying home again after extended leave and being isolated from everyone has lead to depression - on top of the extreme fear of my … baby contracting the virus.” Working from home likewise resulted in erosion of social support from co-workers.

Some nurses and physicians felt ostracized by the community due to fear of contracting the virus, combined with perceived empty gestures of support: “… friend, neighbors considering me a deadly weapon because I might be infected and a threat to them.” Difficult situations arose when nurses and physicians felt that their ability to communicate risk of the pandemic with their patients was superseded by misleading media coverage, resulting in self-censorship, which is a form of social isolation: “It’s hard to do my job when families are constantly throwing cnn[sic] or google in my face! … This histeria[sic] has foced[sic] me to socially distant or i[sic] get social[sic] shamed.”

#### Stressed colleagues

Give the importance of teamwork to successful provision of healthcare, perceived stress of co-workers proved difficult: “Staff breaking down and crying”. For some, breakdown in teamwork manifested in multiple ways, anchored in worries about risk of infection and unequitable workloads, and aggravated by lack of administrative support.

Several nurses and physicians appeared find it difficult to deal with colleagues who appeared to not be bearing well under what was perceived as normal pressures of intensive care: “Mostly hearing co-workers ‘fishing for thank you’s’ [sic]. … being overly dramatic”. It appears that difficulties related to perceived “irrational fears” were conflated with frustration about how the pandemic was portrayed in the media and politicized.

#### Aggression and anger

Encounters with agitated, angry and aggressive patients and their families was among the most adverse experiences of the pandemic for some: “Being yelled and screamed at by distraught, isolated suspected and confirmed Covid-19 patients because they did not feel I was moving fast enough … .”

Some nurses and physicians were distressed by their own anger.

#### Uncertainty

Uncertainty of the impact of the virus on one’s patients intermingled with sense that patients are not receiving the best possible care: “Every day feeling like I am failing my patients because we don’t know enough … .” The sense of apprehenssion troubled some: “Feeling that I will encounter situations that I am not confident in my ability to manage.” The uncertainty about what holds true about the pandemic made it difficult for some to train staff “on policies/initiatives that [one does] not personally agree with.” Some nurses and physicians reported that “managing staff anxiety, fear” was particularly stressful in light of “knowledge deficits”. The constant changes, “at a daily and sometime hourly basis,” in the understanding of the pandemic at the leadership level precipitated stressful changes in procedures at the bedside. For some, the diversity of views of the pandemic by the public and patients placed nurses and physicians in the crossfire of the competing narratives, such that the community’s uncertainty had an adverse spill-over effect on the frontline nurses and physicians.

#### Poor transparency at work

Some of the most difficult experiences related to “lack of communication”, including “no support from manager regarding how to find information or what to tell patients”. When practice guidelines appeared to make no sense and work-related requests were not perceived as having been dealt with rationally and respectfully, distressing situations arose: “… being told I did not need to wear PPE. After push back, getting the required PPE and being questioned why I needed them.” For some there was an overall sense of “the disorganization, poor communication, and frequent disrespect by [leadership/management]” which included perception of lack of concern for well-being of nurses and physicians: “Being told we are ‘heroes’ while being treated like second class citizens.”

#### Lacking administrative support

An issue closely related to poor communication is perception of lack of administrative support, even among those who otherwise welcomed the challenges of the pandemic:

“I enjoy a challenge, so the pandemic was an opportunity to prove that I can survive and be a positive support for others. The most stressful event so far is witnessing the poor leadership choices. Very disorganized.”

When frontline works appeared to be overlooked in recognition by the management despite taking risks and speaking up in an attempt to remedy lapses in practice, this proved “stressful”: “we were constantly exposed and not recognized at all.” Worse still was “being disciplined for being frustrated with the increased work loaf [sic]”, leading some to believe that they are “unable” to perform their duties both safely and well.

Perceived lack of empathy and “respectful conversation” with administration was led to “unnecessary frustration”: “the disorganization, poor communication, and frequent disrespect by my director and the command center.” When there was perception that rules were not fair, frustration arose due to: “… being ridiculed for wanting to follow set guidelines”. “Lack of empathy and understanding” from leadership appears to be recurring theme in situations that caused hardships to nurses and physicians, for example “when daycare closed and I had to figure out a schedule to still be able to work while my husband, who is also essential, was still able to work and our child was still cared for every day.” One of the expressions of this lack of empathy appears to have been seen in reports that “system is more worried about their bottom line than they are about patient safety”.

#### Childcare

Loss of childcare and the resulting need to balance professional duties as an “essential” worker with family responsibilities was among most difficult experiences of the pandemic reported by those with children living at home: “Working full time … while the schools have been closed for the last two months, my [children] are at home …”. This was especially difficult when person was the only available caregiver.

### Themes of “What has been the event that has most reinforced your pride in your professional behavior?”

Frequencies and names of the coded themes of the events since the start of the pandemic that reinforced pride in the profession are captured in Table 2. They also indicate our perceived association of these themes with autonomy-supportive factors experienced by nurses and physicians. The nuances and meaning of the coded themes are illustrated blow using the respondents’ own words, with detailed presentation of representative quotations in Supplemental Material B.

#### Courage, composure under pressure

It is not surprising that individuals who felt that they conquered their fears and remained on the job were proud of the fact: “… I haven’t allowed COVID to overtake my life with fear/anxiety”. Some were proud not only of the fact they continued to work but that they could say: “I still love the work I do”. The pride in overcoming personal fears was related to putting the needs of patients above personal risks: “Showing up at work everyday [sic], odd shifts despite the hardships of felling unprepared without all of the answers and constraints on family life… .” For some, the pride in steadfastness of nurses and physicians intertwined with pride in effective teamwork: “The resolve of the … doctors and nursing staff …. There is a collegial feeling of purpose that is palpable but hard to articulate.” Some were proud of being up to the challenge of the pandemic as the team: “We succeeded in spite of administration. Not because of it.” This sense of autonomous professional success is summarized as “relying On myself”. Some noted that they were proud that their “… confidence has grown throughout this whole experience … .”

#### Volunteering for hazardous tasks and altruism

One example of courage under duress was volunteering for tasks perceived to be hazardous, an understandable source of personal pride: “I will see covid patients to keep another provider, at higher risk that I”. Some nurses and physicians were proud of doing what they thought was right despite acknowledging personal risks: “Being able to be there for the community and treat patients while risking my own self and family’s health.”

Some reported being proud of taking extra effort to do what they thought was right, even when there was perceived lack of support for such acts: “… had to use community resources and also my own money to buy [N95 masks and face shields]…. This was both very stressful and proud moment.” Altruism and courage displayed by others was a clear source of pride about the profession overall.

#### Quality of care, positive outcomes

Among most common experiences that reinforced pride in the discipline among nurses and physicians was that “the few who have been the most sick with covid and survived”. Beyond survival, specific aspects of patient care that were highlighted among those that instilled pride included “successfully educating patients about what we currently know and easing their fear and anxiety”. Pride in compassionate “high quality of empathetic, therapeutic care” was evident, especially needed due to isolation of patients precipitated by infection control measures: “being able to make these isolated pts smile …”. Success in infection control was a source of pride: “Knowing that I have come to work through this all and have not contracted Covid.” Some shared that they were proud of being able to help others provide the high-quality care.

Examples of assertiveness and successful advocacy on behalf of the patients were a source of pride: “I am proud of my ability to advocate for suffering patients … .” A related source of pride is how nurses and physicians adapted to caring for patients, growing in both confidence and skill required to practice their profession under the pandemic’s constraints; there was a sense that this is what the profession was meant to do: “We were the first COVID unit. Watching the nurses go from being uncertain, scared to totally embracing it and doing an excellent job caring for these patients. Putting patients first. This is our calling, it’s why we are nurses.” Some shared that their pride rested on having provided quality care despite lack of recognition and support: “… we didn’t do it for the recognition anyway, but for our patients.” For many of the respondents, pride arose simply from doing their usual work well despite pandemic.

#### Comforting dying patients

Despite death of patients being named among most difficult experiences by nurses and physicians, the manner they handled death of patients was a source of pride for many: “None of My patients died alone. I was there for each of them.”

#### Innovation and technological sophistication

Some reported overcoming challenges posed by the infection control protocols, reporting that they were proud of: “coming up with out of the box ideas … to facilitate better communication with patient and caregiver.” The fact that developing new way of providing care necessitated initiative due to (perceived) lack of organizational support was a source of pride for some.

#### Community outreach beyond clinical duties and support from community

Some respondents were proud of the fact that they contributed to management of the pandemic outside of their immediate clinical duties: “… making sure I have researched facts I provide to friends/family/patients.”

For some, pride stemmed from recognition of nurses and physicians by the wider community: “The outpouring of community support, donations, thank yous[sic], and the complete strangers that were kind enough to make scrub hats for us.” Recognition of difficulties faced by nurses and physicians by media appeared to be an important part of instilling a sense of pride: “… the news reporting how medical staff and nurses are treated when we try to ourselves safe and healthy by advocating for ourselves and speaking up for what is happening that is not right.” Expressions of gratitude and concern that had a personal touch appeared to be appreciated such as when a former patient called: “… to ask if the nurses are ‘OK’ … .” Expressions of support and gratitude from family members were likewise a source of pride: “My family supporting me in continuing my job as a nurse, despite being away from home, while friends were telling me to get out of nursing due to COVID.”

#### Adaptability and flexibility

Nurses and physicians reported to be proud of how they and their colleagues adapted: “Everyone’s resourcefulness and support in coming up with ways to continue to take care of pts in ways that keep exposure to a minimum.” For some, adaptability that they were proud of appeared to be facilitated by effective teamwork: “Teamwork, sacrifice, and agility of each member of our staff to re-organize and implement provision of care while keeping patients and staff safe as possible.”

#### Revealing leadership qualities

One respondent was proud of how they “took it upon” themselves “to guide and take care of” their “team during time where office manager lacked decision making and / or communication with staff”. Some found effective leadership displayed by others a source of pride for the profession as a whole: “… the course shown by some nursing, physician and several other staffs.” Those in the leadership positions appreciated being recognized when they proved effective.

#### Teamwork

Teamwork was a source of pride in how some nurses and physicians comported themselves, despite fears and uncertainties. This is exemplified by a report “that nurses and other health care providers, even when scared without having information, still came to work and did their job for their patients.” The sense of *esprit de corps* and focus on the mission to serve patients despite personal risk was on display when “ nurses and doctors work expertly toward one mission, saving the life before you while keeping all stakeholders involved in the care safe from harm and illness. I admire their ability to set aside their fears and go into action, regardless of their internal insecurities.” The pride in teamwork arose because it entailed peer support: “Happy with support of co-workers at coming together to work in a pandemic.”

#### Gratitude from patients

The sense of professional pride was reinforced when patients expressed gratitude: “compliments from my patients, when they tell me they appreciate me listening to them and taking the time needed to take great care of them”. It appears that gratitude from some patients and their families was related to both quality of care and recognition of personal risks that nurses and physicians endured in order to care for the patients.

#### Recognition from administrators and leadership of healthcare systems

Nurses and physicians who believed that they were appreciated by their employers and team leads tended to report this as one of the experiences that reinforced pride in their professions. When organizations did not come across as divided between nurses and physicians and administration, there was a reason to report this as a proud achievement: “The love, support and spirit of my nurses, first responders, and hospital administration.” For some nurses and physicians, the pride in their professions was reinforced by acts of advocacy by those in a position to do so: “my union’s persistence”.

#### Not proud

Some respondents elected to tells us why there were not proud of their profession, even though we did not inquire about this. Some regretted not having done enough: “I should have tried harder to get the necessary resources … I should not have let the counseling deter me for advocating for myself and my colleagues.” The sense of not being able to advocate for the best handling of the pandemic appears to be a common them among those who appear to feel less than proud about their profession’s contribution during pandemic, with the source of frustration aimed at government and hospital leadership. Others expressed dismay at how healthcare systems reacted, conveying a sense of futility and apathy in light of a contradiction between known best practices and demands of administrators: “I try to keep it together for my patients, but who cares about PPE regulations … ? We know this is wrong, but our administration tells us to do it anyway- so why try?” Some responses appear to capture a sense of exhaustion, burn-out: “I don’t[sic] have any feelings any more to my profession. … Having feelings is not much use.”

It appears that for some respondents pride in the profession depended on action of external forces and how they were treated rather than intrinsic values or achievements: “We have been treated increasingly worse as this rolls on. Most staff are barely hanging on and several have quit.” It would appear that for some the value of recognition within organizations and faith in rational actions in the best interest of patients outweighs positive impact of support from community at large: “Media calls us heroes while leadership treats us like an expendable commodity. … MANY patients and staff exposed due to incompetent decisions … .”

## Discussion

We believe that our contention that autonomy-supportive experiences lead to positive perception of the pandemic and better mental health is borne out by thematic analysis and association of the elicited themes with measures of anxiety and depression. For example, we observed that experiences that undermined the sense of competence, such as in performance of clinical duties, were associated with anxiety. Breakdown in communications and administrative support at work appeared to undermine the sense of autonomy (e.g., instructions constantly changing without justification) and relatedness (e.g., administration working against nurses and physicians), and correlated with both greater anxiety and depression. Pride in technological sophistication and inventiveness were indications of sense of autonomy and competence, lessening anxiety. A sense of pride that emanated from community support can be seen as support for sense of relatedness and reduced depression. Against our hypothesis, volunteering for hazardous tasks, which can be seen as expression of both autonomy (i.e., person volunteered) and relatedness (e.g., motivated by desire to protect co-workers) was associated with heightened anxiety and depression. It is perhaps persons who are proud of such experiences who need to be targeted for mental health supports, in addition to those who struggled with distressing events. Overall, any distressing events were related to both anxiety and depression but reports of events that instilled pride counter-acted risk of depression. This again suggest that *fostering a sense of pride in how one comports themselves may an effective means to safeguarding against depression and anxiety during disasters*.

Our findings must be placed in the context of the US healthcare system. Nurses and physicians in the US are trained to be independent practitioners. This means they assess patients, make decisions as to what care or treatment is called for, and then develop a plan with the patient and their family. During the COVID-19 pandemic in the US, as these nurses and physicians struggled to develop a plan for care, because the promising practices of treatment were still in development, with ever-changing and conflicting US CDC recommendations mirrored in hospital policies. This caused stress on the healthcare workers who were used to *independently developing and implementing plans of treatment* without interference from government or hospital administration. With the national management of the COVID-19 pandemic, this was not the case, and we hypothesize that this led to the frustration, anger, disappointment, anxiety, and lack of individual pride in practice. For the future, policymakers may respect practitioners, encourage them to innovate during crises, thereby helping them be proud of their work, and encouraging them to improve clinically relevant practice.

The narratives captured patient experiences during COVID-19 pandemic, which was not among our aims, and concerns of nurses and physicians about quality of care (correlated with anxiety). *Did nurses and physicians advocate for their patients when asked about their own experiences of the pandemic?* It can be inferred from distressing experiences of nurses and physicians, that infection controls that prevented families from being with dying patients should have been altered to be more humane. Likewise, the narratives imply a question as to whether mental health support and communication protocols were in place to supply best possible patient care. We think that these results can shape policies that are beneficial to both nurses and physicians and patients.

Themes of most difficult events among nurses and physicians overlapped with those elicited in a sample of general population in Philadelphia, where TH is located, over a similar time period as this report [18]. Specifically, the themes shared across samples were economic woes, disruption of working lives, childcare struggles, worries about health (including contracting COVID-19), uncertainty, media coverage, and frustration with government response. Themes associated with mood disorders that appear common to the two samples were worries about contracting COVID-19 and experiencing inconsistent messages and poor support from those perceived to be positions of power (respectively: hospital administration vs. government), implying that they are not specific to nurses or physicians, and may be related to universal psychological needs articulated by the SDT.

Our work suffers from numerous limitations arising from cross-sectional design, even though we did control for health history and demographics. Notably, principal component analysis shows that both difficult and proudful experiences are not exclusively clustered among persons with history of mood disorders. However, all data is self-reported, thus being subject to biases from social desirability and correlated errors. Lack of responses in shared narratives cannot be interpreted as absence of relevant events and our conclusions are thus tempted by bias due to willingness to share experiences. Some of the experiences, such as those arising from management environment and economic situation must have been shared by all respondents within healthcare systems and professions, but only reported by some. Therefore, our conclusions are limited to perception of events and willingness to share them; we mitigated bias from shared working conditions by controlling for healthcare system and profession in statistical models. We lacked some of the information that would have been helpful in interpreting data, such as availability and utilization of mental health supports. We only studied two healthcare systems and struggled with (typically) low participation rates, undermining generalizability of the findings.

Despite limitations, our work offers valuable insights and can help manage mental health challenges experienced by nurses and physicians during response to epidemics. For example, the elicited narrative themes of the most difficult or distressing events and moments that instilled pride in the professions can be the foundation of a survey instrument on perception of pandemics. This may assist in monitoring wellbeing of nurses and physicians during response to emergencies and obtaining their buy-in with changes in care provision. It is plausible that feedback from nurses and physicians to leadership that is inherent in autonomy-supportive buy-in would improve patient care, given that nurses and physicians were proud to share their innovative solutions to challenges of patient care during the pandemic. We note that some themes are not specific to infectious disease outbreaks but rather speak to autonomy-supportive workplace practices and leadership in general. Such practices and leadership can be fostered within healthcare systems and evaluated. In particular, autonomy-supportive leadership is suggested to be related to improved well-being in a recent meta-analysis [19]. It was discussed how leaders among nurses can improve mental health of other nurses during COVID-19 pandemic [20]. Additionally, it is indicated that during COVID-19 pandemic specifically, autonomy-supportive environment anchored in perceived social responsibility of the employer (but not extrinsic motivation) produced higher performance among employees, presumably in part by protecting their mental health under duress of pandemic-precipitated disruptions [21]. There is limited information on what worked (and did not) in helping alleviate mental health distress during the pandemic among healthcareworkers, but Wang et al.,[5] report the benefits of “positive refocusing” among nurses in China.

## Conclusion

Our findings document the broad spectrum of difficult and positive experiences of some nurses and physicians during the first wave of COVID-19 pandemic. We offer insights into how to monitor healthcare workers’ well-being during pandemic and provide evidence that autonomy-supportive policies may foster well-being under duress.

## Electronic supplementary material

Below is the link to the electronic supplementary material.

Supplementary Material 1

Supplementary Material 2

## Data Availability

The researchers cannot share data publicly due to the data privacy rules that were the basis of the ethics approval for this study. Data supporting the results reported in this study are kept in a locked password protected repository on a Drexel University owned server and must be kept confidential to protect individuals’ privacy and as per the Institutional Review Boards. De-identified summaries of the data necessary to either reproduce specific results, or illuminate questions not addressed in the paper, will be shared upon reasonable request to the corresponding author.
