# Supplementary material for: Pride and adversity among nurses and physicians during the pandemic in two US healthcare systems: a mixed methods analysis
Source: BMC Nurs. 2022 Nov 9;21:304. doi: 10.1186/s12912-022-01075-x (PMC9643994; doi:10.1186/s12912-022-01075-x)

**Supplemental Material A**

**Table A1:** Eigenvectors of principal components (Prin) examining the inter-relationship of binary reports of distressing experiences, experiences that reinforced pride in the profession, Hospital Anxiety and Depression Scores (HADS) at the time of data collection that indicate case of anxiety or depression, history of anxiety or depression prior the COVID-19 pandemic, and reports of treatment year before pandemic for anxiety or depression.

|  | | | | |
| --- | --- | --- | --- | --- |
|  | **Prin1** | **Prin2** | **Prin3** | **Prin4** |
| **Distressing experience reported (distress)** | 0.068 | 0.690 | -0.085 | 0.216 |
| **Experiences that reinforced pride reported (proud)** | 0.030 | 0.688 | -0.181 | -0.113 |
| **HADS Anxiety score ≥11 (case) (HADSA11)** | 0.286 | 0.148 | 0.615 | -0.708 |
| **HADS Depression score ≥11 (case) (HADSD11)** | 0.259 | 0.066 | 0.668 | 0.656 |
| **History of anxiety or depression (AnxDepHx)** | 0.526 | -.0189 | -0.236 | -0.062 |
| **Anxiety treatment year before pandemic (AnxT)** | 0.537 | -0.112 | -0.203 | 0.052 |
| **Depression treatment year before pandemic (DepT)** | 0.530 | -0.108 | -0.197 | 0.049 |

**Figure A1:** Component factor pattern profiles from the principal components analysis with eigenvectors from Table A1 (se for variable definitions).


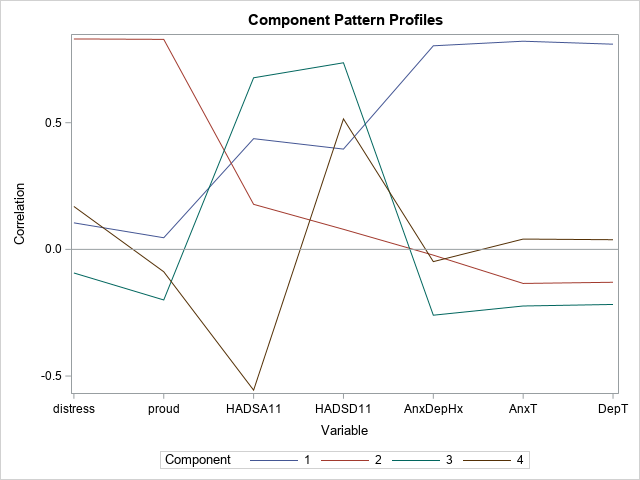

Supplement: Supplementary file 1 — Supplementary Material 1 [file 12912_2022_1075_MOESM1_ESM.docx]
